# Facteurs de risque de l'infection par le VIH dans le district de santé de Meyomessala au Cameroun

**DOI:** 10.11604/pamj.2014.18.161.3238

**Published:** 2014-06-19

**Authors:** Francois-Xavier Mbopi-Keou, Georges Nguefack-Tsague, Ginette Claude Mireille Kalla, Stéphanie Abo'o Abessolo, Fru Angwafo, Walinjom Muna

**Affiliations:** 1 Faculté de Médecine et Sciences biomédicales, Université de Yaoundé I, Yaoundé, Cameroun; 2 Laboratoire National de Santé Publique, Ministère de la Santé Publique, Yaoundé, Cameroun; 3 Centre Hospitalier et Universitaire de Yaoundé, Cameroun; 4 Hôpital Gynéco- Obstétrique et Pédiatrique de Yaoundé, Cameroun

**Keywords:** VIH, facteurs de risque, maladies sexuellement transmissibles, Meyomessala, Région du Sud, Cameroun, HIV, risk factors, sexually transmitted diseases, Meyomessala, South region, Cameroon

## Abstract

**Introduction:**

L'objectif de ce travail était de déterminer les facteurs de risque de l'infection par le VIH dans le district de santé de Meyomessala (Région du Sud) au Cameroun.

**Méthodes:**

Il s'agissait d'une étude transversale, descriptive et analytique qui s'est déroulée de Février à Mai 2011. Pour cette étude, nous avons obtenu une clairance éthique.

**Résultats:**

L’échantillon était constitué de 315 participants dont 181 (57,46%) hommes et 134 (42,54%) femmes. L’âge moyen était de 24,5±8ans (extrême: 15-45ans). Quarante personnes (40) étaient séropositifs, soit une prévalence de l'infection par le VIH de 12,7%. Cette prévalence augmentait significativement (p = 0) avec le nombre de partenaires occasionnels au cours des douze derniers mois, allant de 2,7% chez ceux n'ayant eu aucun partenaire occasionnel à 21,25% chez ceux ayant plus de trois partenaires occasionnels (RC = 9,72; IC = 1,27-74,14; P = 0,03). le fait d’être âgé entre 20 et 24 ans (RC = 4,88; IC = 1,74-13,67; p = 0), avoir plus de trois partenaires sexuels au cours des douze derniers mois (RC = 9,72; IC = 1,27-74,14; p = 0,03), avoir les rapports sexuels avec les prostitués (RC = 2,86; IC = 1,42-5,76; p = 0), avoir eu le chlamydia (RC = 3,00; IC = 1,07-8,39; p = 0,04), avoir eu la syphilis (RC = 3,35; IC = 1,57-7,14; p = 0), avoir des avantages sociaux lors du premier rapport sexuel (RC = 2,57; IC = 1,03-6,43; p = 0,04) constituaient des potentiels facteurs de risque du VIH.

**Conclusion:**

Il apparait urgent d'intensifier les campagnes de sensibilisation au risque d'infection par le VIH et les maladies sexuellement transmissibles dans le district de santé de Meyomessala

## Introduction

L'infection à VIH représente de part le monde un problème majeur de santé publique [1] en dépit les grandes interventions menées à l’échelle planétaire. Au Cameroun, la prévalence de l'infection par le VIH a connue une progression constante [2–6], même si des récentes enquêtes ont démontré un ralentissement de la courbe d’évolution de l’épidémie [4, 7]. Le déploiement des moyens de lutte contre cette terrible maladie dans l′objectif de son éradication ou à tout le moins de sa réduction substantielle est révélateur de la crainte que suscite cette pandémie, mais aussi du degré de prise de conscience de la menace qu′elle inspire.

Avec 560 000 personnes vivant avec le VIH et plusieurs souches virales, le Cameroun est plus qu′un foyer de la pandémie [1–3], c′est un laboratoire par excellence d′expérimentation, tant de l′évolution de la pandémie, que des stratégies de lutte. Les progrès thérapeutiques ont permis au cours des dernières années d′influer sur l′histoire naturelle de la maladie en réduisant la morbidité de 80%, et l′évolution vers le stade de SIDA maladie [8]. Les stratégies antirétrovirales, quelque soit leur efficacité, ne permettent pas une éradication du virus de l′organisme. Le moyen de lutte le plus efficace reste la prévention. Les méthodes préventives (dépistage et conseil) concourent à la prise en charge rapide et adéquate des personnes infectées et à la conscientisation des personnes séropositives et séronégatives sur les comportements à risque. La présente étude entend s′inscrire dans la logique générale d′appréciation des résultats obtenus, à partir d′une nouvelle lecture de la prévalence et des facteurs de risque dans certaines localités urbaines ou rurales. Aussi, nous avons choisi le district de santé de Meyomessala qui réunie les caractéristiques d′un espace urbain et rural, notre objectif étant de déterminer les facteurs de risque de l'infection par le VIH dans la dite localité.

## Méthodes

Il s'agissait d′une étude transversale, descriptive et analytique ayant eu lieu dans la commune de Meyomessala (Région du Sud Cameroun) de février à mai 2011. Etait inclue dans l’étude toute personne résidant à Meyomessala, âgée d′au moins 15 ans et possédant les résultats d'un test de VIH datant d'au plus 2 mois avant le début du recrutement.

La taille minimale de l’échantillon avait été calculée avec un seuil d'erreur de 5%, selon la formule suivante [9]:

**N= (P(1-p)(Z_α/2_)^2^)/d^2^**

P: Prévalence de l'infection à VIH au Sud du Cameroun = 6,5% [4, 7]; **Z_α/2_**: Valeur du fractile de la distribution normale à 5%= 1,96; d: Degré de précision = 3%; la taille calculée est de N = 260 personnes. En tenant compte d'un possible taux de non réponse de 21% (soit 55 personnes), la taille de l’échantillon minimale était de 315 personnes.

Le recrutement s'est fait tous les jours pendant la période de l’étude dans 6 villages tirés au sort (sondage aléatoire simple) parmi 102 dans la commune de Meyomessala. Le Tableau 1 montre le nombre d'enquêtés par village, calculé proportionnellement à la population de chaque village. Les informations ont été recueillies auprès des participants à l'aide d'un questionnaire conçu et pré-test à cet effet. Les recrutements se sont faits dans les centres de santé des différents villages choisis. Nous avons obtenu le consentement éclairé de tous les participants à l’étude. Ces derniers ne risquaient pas de subir un quelconque préjudice s′ils n'acceptaient pas de participer à l’étude ou si pour une raison ou une autre ils décidaient au courant de l’étude de se retirer. En outre, pour cette étude, nous avons obtenu une clairance éthique.

**Tableau 1:** Nombre d'habitants enquêtés par village

Village	Population	Pourcentage (%)	Taille échantillon
Bengbis	4 150	20,85	66
Goasseu	1 100	5,53	17
Koum yetotan	1 845	9,27	29
Mbometa'a	4 300	21,60	68
Mengon	4 616	23,19	73
Meyomessala	3 896	19,57	62
Total	19 907	100,00	315

Les données collectées ont été saisies et analysées en utilisant les logiciels Epi-Info version 3.5.3 et IBM SPSS version 19. Les variables quantitatives étaient représentées par la moyenne ± écart-type; et les variables qualitatives par des pourcentages (%). Les différences entre les proportions ont été analysées en utilisant des tables de contingence et en appliquant le test de Chi-deux de Pearson et le test exact de Fisher lorsque les effectifs attendus dans une cellule au moins étaient inférieurs à 5. L'ampleur de l'association entre deux variables qualitatives a été évaluée par le rapport de côte (RC), avec un intervalle de confiance à 95%, pour déterminer les facteurs de risque pris individuellement (analyse univariée). Le test de Cochran-Armitage [10, 11] a été utilisé pour déterminer l'existence d'une variation linéaire de proportions en fonction d'une variable ordinale. Afin de tenir compte d’éventuels facteurs de confusion, la méthode de sélection par rapport de vraisemblance ascendante a été utilisée pour sélectionner les variables à inclure dans un model logistique binaire multiple (analyse multivariée). Les valeurs p

## Résultats

La population d’étude était constituée de 315 participants, dont 57,46% d'hommes et 42,54% de femmes. L’âge moyen était de 24,5±8ans (extrême: 15-45ans). Le Tableau 2 montre que la classe d’âge la plus représentée était celle de 15 à 19 ans (31,75%). Les célibataires étaient majoritairement représentés (77,78%). Le niveau secondaire avait été atteint par 70,16% des participants. La plupart de nos participants 44,76% pratiquait le catholicisme. Plus de 6 personnes sur 10 (62,53%) étaient du Sud Cameroun. Quarante (40) personnes étaient séropositifs, soit une prévalence de VIH de 12,7%.

**Tableau 2:** Prévalence du VIH selon certaines caractéristiques sociodémographiques

Caractéristiques	VIH +	VIH-	Prévalence (%)	Effectif	Ensemble (%)	RC (95%IC)	P-RC
**Sexe** (P-Test de Chi-Deux = 0,5)						
Homme	21	160	11,6	181	57,46	0,79 (0,40-1,55)	0,5
Femme*	19	115	14,18	134	42,54	1,00	
**Age** (P-test de trend de Cochran-Armitage = 0,25)						
15-19*	5	95	5	100	31,75	1,00	
20-24	19	74	20,43	93	29,52	4,88(1,74-13,67)	0,00
25-29	5	43	10,42	48	15,24	2,21(0,61-8,03)	0,23
30-34	5	21	19,23	26	8,25	4,52(1,20-7,04)	0,03
35-39	3	26	10,34	29	9,21	2,19(0,49-9,78)	0,30
40-45	3	16	15,79	19	6,03	3,56(0,77-6,39)	0,10
**Statut Matrimonial** (P-Test exact de Fisher = 1)						
Célibataire*	33	212	13,47	245	77,78	1,00	
Marié	6	45	11,76	51	16,19	0,86(0,34- 2,17)	0,74
Séparé	0	6	0	6	1,9	1,07(0,12-9,18)	0,95
Divorcé	1	7	12,5	8	2,54	0,92(0,11-7,70)	0,94
Veuf/veuve	0	5	0	5	1,59	1,28(0,15-1,34)	0,82
**Profession** (P-Test exact de Fisher = 0,20)						
Elève*	17	127	11,8	144	45,71	1,00	
Fonctionnaire	3	49	5,8	52	16,51	0,46(0,13-1,63)	0,23
Prive	5	31	13,88	36	11,43	1,20(0,41-3,52)	0,73
Sans emploi	15	68	18,07	83	26,35	1,65(0,78-3,50)	0,19
**Education** (P-test de trend de Cochran-Armitage = 0,03)						
Aucun*	0	7	0	7	2,22	1,00	
Primaire	16	47	25,4	63	20	2,38(0,27-20,89)	0,43
Secondaire	22	199	9,95	221	70,16	0,77(0,09-6,58)	0,81
Supérieur	2	22	8,33	24	7,62	0,64(0,05- 8,12)	0,73
**Religion** (P-Test exact de Fisher = 0,80)						
Catholique	18	123	12,85	141	44,76	2,20(0,27-17,64)	0,46
Protestant	16	124	11,34	140	44,44	1,94(0,24-15,65)	0,54
Musulman	3	15	16,67	18	5,71	3,00(0,28-32)	0,36
Autres églises*	1	15	6,25	16	5,09	1,00	
**Région** (P-Test exact de Fisher = 0,99)						
Centre	5	39	11,36	44	13,97	1,15(0,25 -5,24)	0,85
Est	1	11	8,3	12	3,81	0,82(0,08-8,73)	0,87
Nord	1	11	8,3	12	3,81	0,82(0,08-8,73)	0,87
Ouest	2	18	10	20	6,35	1,00(0,15-6,59)	1,00
Sud	28	169	14,21	197	62,53	1,49(0,42-5,25)	0,53
Autre*	3	27	10	30	9,52	1,00	

* Niveau de référence; RC = Rapport des Cotes; 95%IC = Intervalle de Confiance à 95%

### Prévalence du VIH selon certaines caractéristiques sociodémographiques de la population d’étude

Le Tableau 2 montre que la prévalence du VIH était plus élevée chez les femmes (14,18%) contre 11,60% chez les hommes (p = 0,5). Cette prévalence était plus élevée dans la tranche d’âge 20-24ans (20,43%); elle n'augmentait pas avec l’âge (p = 0,25). Les célibataires étaient les plus touchés (13,47%), suivis des mariés (11,76%), et des divorcés (12,50%); différences non statistiquement significatives (p = 1). Les sans-emplois avaient une prévalence du VIH plus élevée (18,08%); suivis des travailleurs du privé (13,88%). On notait une baisse significative (p = 0.03) de la prévalence du VIH avec le niveau d’éducation, allant de 25,4% au primaire à 8,33% au supérieur. Il n'y avait pas de différence significative entre les religions, bien que les musulmans avaient une plus grande prévalence de l'infection par le VIH (16,67%). Les personnes originaires de la région du Sud étaient les plus touchées (14,21%), différences non significatives (p = 0,99).

### Prévalence du VIH selon certains comportements sexuels

Le Tableau 3 montre que plus de 4 personnes sur 10 (45,08%) avaient eu leur premier rapport sexuel avant l’âge de 16 ans; 13,01% les avaient eus à 20 ans et plus. La plupart des enquêtés (69,52%) avaient des partenaires occasionnels; et 45,68% avaient plus de 3 partenaires. Plus de 8 participants sur 10 (81,9%) avaient déjà utilisé le condom au moins une fois au cours de leur vie, et 62,22% l'avaient utilisé au cours des douze derniers mois; 21,59% d'individus avaient eu des rapports sexuels avec des prostituées.

**Tableau 3:** Prévalence du VIH selon certains comportements sexuels

Comportement sexuel	VIH +	VIH-	Prévalence (%)	Effectif	Ensemble (%)	RC (95%IC)	P-RC
**Age aux premiers rapports sexuels** (P-test de trend de Cochran-Armitage = 0,29)							
<16*	15	127	10,56	142	45,08	1,00	
16-17	11	74	12,94	85	26,98	1,26 (0,55-2,88)	0,59
18-19	8	39	17,02	47	14,92	1,74(0,69-4,40)	0,24
20 ans et plus	6	35	14,63	41	13,02	1,45 (0,52-4,02)	0,47
**Nombre de partenaires sexuels au cours des 12 derniers mois** (P-test de trend de Cochran-Armitage =0,000)							
0*	1	36	2,7	37	11,75	1,00	
1	5	81	5,65	86	27,30	2,22 (0,25-19,71)	0,47
2	8	57	12,3	65	23,38	5,05 (0,61-42,10)	0,13
3 et plus	27	100	21,25	127	45,68	9,72 (1,27-74,14)	0,03
**Utilisation du condom** (P-Test de Chi-deux = 0,59)							
A déjà utilisé un condom*	6	51	10,52	57	18,10	1,00	
N'a jamais utilisé de condom	34	224	13,18	258	81,90	1,29 (0,51-3,24)	0,59
**Utilisation du condom lors des derniers rapports sexuels au cours des douze derniers mois** (P-Test de Chi-deux = 0,08)							
Oui	10	109	8,4	119	37,78	1,00	
Non	30	166	15,31	196	62,22	1,97(0,93-4,19)	0,08
**Rapports sexuels avec une prostituée** (P-Test de Chi-deux = 0,00)							
Oui	16	52	23,53	68	21,59	2,86 (1,42-5,76)	0,00
Non*	24	223	9,72	247	78,41	1,00	

* Niveau de référence; RC = Rapport des Cotes; 95%IC = Intervalle de Confiance à 95%

La prévalence du VIH augmentait significativement (p = 0) avec le nombre de partenaires occasionnels au cours des douze derniers mois, allant de 2,7% chez ceux n'ayant eu aucun à 21,25% chez ceux ayant plus de trois. En effet, ces derniers avaient près de 10 fois plus de risque d’être séropositifs que ceux n'ayant eu aucun partenaire sexuel occasionnel (RC = 9,72; IC = 1,27-74,14; P = 0,03). Il n'y avait pas de différence significative entre l’âge au premier rapport sexuel et la prévalence du VIH (p = 0,29). Ceux qui n'avaient pas utilisé le condom lors des derniers rapports sexuels au cours des douze derniers avaient une prévalence du VIH de 15,31% contre 8,40% pour ceux ayant utilisé (p = 0,08). Elle était aussi élevée chez ceux ayant eu des rapports avec des prostituées (23,53%); ces derniers avaient près de trois fois plus de risque d’être infecté par le VIH que ceux qui n'avaient pas eu de rapports avec les prostitués (OR = 2,86; IC = 1,42-5,76; P = 0).

### Prévalence du VIH selon le type d'infection sexuellement transmissible (IST), consommation d'excitants et circonstance du premier rapport

Il ressort du Tableau 4 que la plupart de nos participants avait eu leur premier rapport sexuel par curiosité (34,92%); ceux ayant eu par viol représentaient 5,71%. La prévalence du VIH était plus élevée chez ceux ayant eu leur premier rapport sexuel par besoins d'avantages sociaux (26,19%); ces derniers avaient près de trois fois plus de risque d’être séropositifs que ceux qui l'avaient eu par amour du partenaire (RC = 2,57; IC = 1,03-6,42; p = 0.04). La plupart des individus (65,71%) n'avaient pas eu d'IST, et 19,68% avaient eu la syphilis. La prévalence de VIH était élevée chez ces derniers (24,19%), suivie du chlamydia (22,20%). Ceux ayant eu la syphilis avaient plus de trois fois plus de risque d’être infecté par le VIH que ceux n'ayant eu aucune IST ((RC = 3,35; IC = 1,57-7,14; p = 0). La majorité des individus consommaient de l'alcool (59,68%), et 3,17% la drogue. Chez ces derniers la prévalence du VIH était de 20% (p = 0,49).

**Tableau 4:** Prévalence du VIH selon le type d'infection sexuellement transmissible (IST), consommation d'excitants et circonstance du premier rapport

Caractéristiques	VIH +	VIH-	Prévalence (%)	Effectif	Ensemble (%)	RC (95%IC)	P-RC
**IST** (P-Test exact de Fisher = 0,0058)							
N'a jamais eu d'IST ni de symptôme*	18	189	8,67	207	65,71	1,00	
Chlamydia	6	21	22,20	27	8,57	3,00(1,07-8,39)	0,04
Syphilis	15	47	24,20	62	19,68	3,35(1,57-7,14)	0,00
Gonococcie	2	14	12,50	16	5,08	1,50(0,32-7,13)	0,61
**Consommation d'excitants**							
**Alcool** (P-Test de Chi-deux = 0,97)							
Oui	24	164	12,76	188	59,68	1,02(0,52-2,00)	0,97
Non*	16	111	12,59	127	40,32	1,00	
**Tabac** (P-Test exact de Fisher = 0,73)							
Oui	5	40	11,11	45	14,29	0,84(0,31-2,27)	0,73
Non*	35	235	12,96	270	85,71	1,00	
**Drogue** (P-Test exact de Fisher = 0,49)							
Oui	2	8	20,00	10	3,17	1,76(0,36-8,58)	0,49
Non*	38	267	12,50	305	96,83	1,00	
**Circonstances du premier rapport sexuel** (P-Test exact de Fisher = 0,22)							
Amour du partenaire*	12	87	12,12	99	31,43	1,00	
Viol	2	16	11,11	18	5,71	0,91(0,19-4,44)	0,90
Curiosité	12	98	10,90	110	34,92	0,89(0,38-2,08)	0,78
Pression de l'entourage	3	23	11,54	26	8,25	0,95(0,25-3,63)	0,94
Avantages sociaux	11	31	26,19	42	13,33	2,57(1,03-6,42)	0,04

* Niveau de référence; RC = Rapport des Cotes; 95%IC = Intervalle de Confiance à 95%

### Prévalence du VIH selon les connaissances

Le Tableau 5 montre que le mode de transmission le plus connu était le rapport sexuel non protégé (89,84%). Par ailleurs on enregistrait respectivement 9,84% et 7,94% d'individus qui pensaient que le VIH pouvait se transmettre par la piqure de moustique et par la salive. L'utilisation du condom (87,94%) représentait le moyen de prévention le plus cité.

**Tableau 5:** Prévalence du VIH selon les connaissances

Caractéristiques	VIH +	VIH-	Prévalence (%)	Effectif	Ensemble (%)	RC (95%IC)	P-RC
**Modes de transmission**							
**De la mère à l'enfant**							
Oui	27	196	12,11	223	70,8	0,84(0,41-1,71)	0,62
Non	13	79	14,13	92	29,2	1,00	
**Par les objets souillés**							
Oui	30	187	13,9	217	68,89	1,41(0,66-3,02)	0,37
Non	10	88	10,2	98	31,11	1,00	
**Par rapport sexuel non protégé**							
Oui	35	248	12,36	283	89,84	0,76(0,28-2,11)	0,60
Non	5	27	15,62	32	10,16	1,00	
**Par transfusion sanguine**							
Oui	33	226	12,74	259	82,22	1,02(0,43-2,44)	0,96
Non	7	49	12,5	56	17,78	1,00	
**Par piqûre de moustique**							
Oui	2	29	6,45	31	9,84	0,45(0,10-1,95)	0,28
Non	38	246	13,38	284	90,16	1,00	
**Par la salive**							
Oui	3	22	12	25	7,94	0,93(0,27-3,27)	0,91
Non	37	253	12,75	290	92,06	1,00	
**Méthodes de prévention**							
**Abstinence**							
Oui	35	227	13,35	262	83,17	1,48(0,55-3,97)	0,44
Non	5	48	9,43	53	16,83	1,00	
**Utilisation de condom**							
Oui	34	243	12,27	277	87,94	0,75(0,29-1,92)	0,54
Non	6	32	15,78	38	12,06	1,00	
**Fidélité**							
Oui	30	225	11,76	255	80,95	0,67(0,31-1,45)	0,31
Non	10	50	16,67	60	19,05	1,00	
**Coït interrompu**							
Oui	2	6	25	8	2,22	2,36(0,46-12,12)	0,30
Non	38	269	12,33	307	97,78	1,00	
**Bain journalier**							
Oui	1	6	14,28	7	2,23	1,15(0,13-9,80)	0,90
Non	39	269	12,66	308	97,77	1,00	

*Niveau de référence; RC = Rapport des Cotes; 95%IC = Intervalle de Confiance à 95%

La prévalence du VIH était plus élevée chez ceux qui pensaient que le virus ne se transmettait pas de la mère à l'enfant (14,13% contre 12,11%), par rapport sexuel non protégé (15,62% contre 12,36%); ces différences ainsi que celles des autres modes de transmission du virus n’étaient pas statistiquement significatives. Il n'existait pas également de différences statistiquement significatives entre les modes de prévention.

**Facteurs associés au VIH dans un model logistique binaire multiple après ajustement par le sexe, âge, statut matrimonial, éducation, nombre de partenaires sexuels, utilisation du condom, infection sexuellement transmissibles (IST) et drogue**

En analyse univariée, le fait d’être âgé entre 20 et 24 ans (RC = 4,88; IC = 1,74-13,67; p = 0), avoir plus de trois partenaires sexuels au cours des douze derniers mois (RC = 9,72; IC = 1,27-74,14; p = 0,03), avoir les rapports sexuels avec les prostitués (RC = 2,86; IC = 1,42-5,76; p = 0), avoir eu le chlamydia (RC = 3,00; IC = 1,07-8,39; p = 0,04), avoir eu la syphilis (RC = 3,35; IC = 1,57-7,14; p = 0), avoir des avantages sociaux lors du premier rapport sexuel (RC = 2,57; IC = 1,03-6,43; p = 0,04) constituaient des potentiels facteurs de risque du VIH.

En analyse multivariée (Tableau 6), les variables sexe, âge, statut matrimonial, éducation, nombre de partenaires sexuels, utilisation du condom, infection sexuellement transmissibles (IST) et drogue; ont été sélectionnées dans le model logistique binaire multiple. Il ressort que être âgé entre 20 et 24 ans (RCa = 6,75; IC = 3,60-15,55; p = 0); être divorcé (e) (RCa = 3,88; IC = 2,04-8,80; p = 0,04), être veuf/veuve (RCa = 5,18; IC = 2,18-12,38; p = 0,04), avoir plus de trois partenaires sexuels (RCa = 10,53; IC = 3,69-25,12; p = 0), n'avoir pas utilisé le condom lors des derniers rapports sexuels au cours des douze derniers mois (RCa = 5,52; IC = 1,98-8,21; p = 0,03), avoir eu le chlamydia (RCa = 4,00; IC = 1,27-10,41; p = 0,03), avoir eu la syphilis (RCa = 4,37; IC = 1,22-9,69; p = 0), et consommer la drogue (RCa = 2,89; IC = 1,78-7,38; p = 0,03); étaient les principaux facteurs associés au VIH.

**Tableau 6:** Facteurs associés au VIH dans un modèle logistique binaire multiple après ajustement par le sexe, âge, statut matrimonial, education, nombre de partenaires sexuels, utilisation du condom, infection sexuellement transmissibles (IST) et drogue

Caractéristiques	RCa	95%RCa	P-RCa
**Sexe**			
Homme	0,55	0,35-1,20	0,33
Femme*	1,00		
**Age**			
15-19*	1,00		
20-24	6,75	3,60-15,55	0,00
25-29	5,38	2,11-10,09	0,01
30-34	4,52	1,10-6,40	0,03
35-39	3,23	1,02-8,45	0,04
40-45	1,15	0,56-5,26	0,15
**Statut Matrimonial**			
Célibataire*	1,00		
Marié	0,45	0,34- 2,56	0,22
Séparé	2,02	0,72-7,16	0,17
Divorcé	3,88	2,04-8,80	0,04
Veuf/veuve	5,18	2,18-12,38	0,04
**Education**			
Aucun*	1,00		
Primaire	0,86	0,10-7,80	0,20
Secondaire	0,52	0,12-1,10	0,06
Supérieur	0,23	0,03- 0,65	0,01
**Nombre de partenaires sexuels au cours des 12 derniers mois**			
0*	1,00		
1	2,42	0,11-7,22	0,15
2	6,18	1,45-13,08	0,03
3 et plus	10,53	3,69-25,12	0,00
**Utilisation du condom lors des derniers rapports sexuels au cours des douze derniers mois**			
Oui*	1,00		
Non	5,52	1,98-8,21	0,03
**IST**			
N'a jamais eu d'IST ni de symptôme*	1,00		
Chlamydia	4,00	1,27-10,41	0,03
Syphilis	4,37	1,22-9,69	0,00
Gonococcie	2,71	0,53-8,27	0,58
**Drogue**			
Oui	2,89	1,78-7,38	0,03
Non*	1,00		

* Niveau de référence; RCa = Rapport des Cotes ajustés; 95%IC = Intervalle de Confiance à 95%

## Discussion

Les facteurs de risque de l'infection par le VIH renvoient en fait à un grand nombre de variables qui interviennent dans l'exposition au VIH, sa transmission et finalement dans la dynamique de l’épidémie [12–21]. Il ressort de différents travaux effectués depuis plus d'une décenie que la transmission du VIH implique la mise en ‘uvre de comportements spécifiques qui exposent un individu au virus [12–21]. Si un individu n'est pas exposé au virus, il ne peut s'infecter. Si un individu exposé ne se protège pas, il a une probabilité d’être infecté. Aussi, des sous-ensembles de déterminants de la transmission du VIH peuvent être déclinés, d'une part en déterminants de l'exposition au virus, et d'autre en déterminants de la transmission elle-même. Ils sont de nature psychosociale ou d'origine biologique.

Dans cette étude, la prévalence globale du VIH était 12,7%. Cette prévalence était plus élevée chez les femmes (14,18%) contre 11,6% chez les hommes. Ce chiffre est le double de celui rapporté dans la région du Sud du Cameroun, et pourrait s'expliquer par l'existence des facteurs de risque identifiés dans cette région tels que la prostitution, la consommation d'alcool et de drogue [4–11]. En outre la présence des plusieurs entreprises et de casernes militaires pourraient contribuer à augmenter cette prévalence [4].

Les femmes étaient les plus infectées par le VIH. Cette vulnérabilité des femmes sur le plan biologique, culturel, économique et social par rapport à l'infection par le VIH a déjà été rapportée [13–19]. La majorité des enquêtés étaient des célibataires (77,8%), ce qui corrobore les travaux antérieurs au Cameroun [4, 12]. La tranche de 20 à 24 ans était les plus touchés par l'infection (20,43%). Ces résultats sont identiques à ceux de l'enquête démographique de santé du Cameroun [4]. En effet, cette tranche d’âge correspond à une période d'activité sexuelle intense qui expose de fait au risque d'infection par le VIH [14, 14].

La prévalence de l'infection par le VIH était plus élevée chez les participants sans emploi. Ce constat revèle une fois l'extrème vulnérabilité de l'individu en Afrique par la pauvreté.

L'entrée dans la sexualité dans notre étude se situant pour la plupart avant l’âge de 16 ans. La prévalence de l'infection par le VIH passait de 10,56% chez les participants ayant eu leur premier rapport sexuel avant l’âge de 16 ans à 17,02% chez ceux qui avaient eu leur premier rapport sexuel entre 18 et 19 ans. L’âge d'entrée dans la vie sexuelle active marque le début de l'exposition au risque d'infection par le VIH [13]. Plus l’âge d'entrée dans la vie sexuelle active est bas, plus le risque d'avoir de plusieurs partenaires est élevé [14]. Hunter et collaborateurs [15] montrent qu'un âge tardif d'entrée dans la vie sexuelle active était associé avec une baisse de risque d'infection.

La prévalence de l'infection par le VIH augmentait avec le nombre de partenaires sexuels; elle allait de 2,7% pour ceux qui n'avaient pas eu de partenaires sexuel au cours des 12 derniers mois à 21,25% chez ceux ayant eu 03 partenaires sexuels et plus. Le multi-partenariat joue un rôle important dans la dynamique de la transmission du VIH [14]. En contexte Camerounais, cette observation corrobore ceux de Rwengue qui rapportait les différences ethniques des comportements sexuels chez les Bamilékés et les Béti [12]. En effet, contrairement aux Bamiléké, les Béti accorderaient très peu d'importance à la virginité et à la fidélité, et encourageraient leurs enfants à mettre au monde un enfant avant le mariage [13].

La quasi-totalité de nos participants avait entendu parler du préservatif (99,68%) mais seulement 62,22% l'avait utilisé au cours des 12 derniers mois. Ceci confirme encore l'existence de barrière à l'utilisation de ce mode de protection. En effet, Il existe encore chez certains un obstacle psychologique à l'utilisation du préservatif, renforcé aussi par un besoin de procréation, d'où l'urgente nécessité de renforcer les campagnes de sensibilisation.

En outre, notre étude démontre que les maladies sexuellement transmissibles, la consommation d'excitant, la prostitution, la pauvreté, les lacunes de connaissances sur les stratégies de prévention et de contrôles de l′infection par le VIH et le fait d'avoir plusieurs partenaires sexuels sont autant de facteurs de risque qui entretiennent la dynamique de l’épidémie dans les pays en développement et notamment en Afrique subsaharienne. Dans cette région du monde, l’épidémie peut être considérée comme la résultante de plusieurs épidémies qui se développent dans des sous-groupes de personnes infectées, chacune ayant sa dynamique propre [16].

Enfin, une des théories les plus importantes de l’épidémiologie des maladies sexuellement transmissibles (MST) est celle du core-group. Selon Plummer et collaborateurs [17], le core-group est une sous-population où chaque individu transmet une MST à plus d'une personne susceptible d’être infectée. Aussi, les prostituées, comme dans notre étude, deviennent des transmetteurs d'autant plus efficaces en Afrique subsaharienne qu'elles n'utilisent pas ou peu de préservatifs, ont de nombreux actes sexuels et un grand nombre de partenaires sexuels, sont souvent infectées par d'autres MST qui facilitent la transmission du VIH et ne connaissent pas en général leur statut sérologique.

## Conclusion

Notre étude démontre à suffire que les maladies sexuellement transmissibles, la consommation d'excitant, la prostitution, la pauvreté, les lacunes de connaissances sur les stratégies de prévention et de contrôles de l′infection par le VIH et le fait d'avoir plusieurs partenaires sexuels sont autant de facteurs de risque qui entretiennent la dynamique de l'infection par le VIH dans les pays en développement et notamment en Afrique subsaharienne [15–21]. Aussi, dans une localité à très forte prévalence de l'infection à VIH comme le district de santé de Meyomessala au Cameroun, il apparait urgent d'intensifier les campagnes de sensibilisation au risque d'infection par le VIH et les maladies sexuellement transmissibles.
